# Supplementary material for: The H3K4 methyltransferase Setd1b is essential for hematopoietic stem and progenitor cell homeostasis in mice
Source: eLife. 2018 Jun 19;7:e27157. doi: 10.7554/eLife.27157 (PMC6025962; doi:10.7554/eLife.27157)
Supplement: Supplementary file 1 — (A) All primers used for genotyping are depicted. (B) All primers used for qRT-PCR are depicted. (se = sense, as = antisense, bp = base pairs) [file elife-27157-supp1.docx]

Supplementary file 1A: **Primers for genotyping**

| Primer pairs | Sequence (5’ – 3’) | Product size (bp) | |
| --- | --- | --- | --- |
| For PCR 1 (presence of *FD* allele) | | | |
| Setd1b_ex5 (se) | GAAACTCGCATGCGCTTCTAC | | 507 bp (*WT*), 696 bp (*FD*) |
| Setd1b_loxP2 (as) | AGTTCATACTGTGGCTGAATGG | |  |
| For PCR 2 (presence of *FDC* allele) | | | |
| Setd1b_flp (se) | GGGTGGAGAGGGAAAGAAAAG | | 1305 bp (*WT*), 1695 bp (*FD*), 390 bp (*FDC*) |
| Setd1b_loxP2 (as) | as above | |  |
| For *Cre* allele | | | |
| Cre_19 (se) | GCCTGCATTACCGGTCGATGCAA | | 726 bp |
| Cre_20 (as) | GTGGCAGATGGCGCGGCAACACC | |  |

Supplementary file 1B: **Primers for qRT-PCR**

| Primer pairs | Sequence (5’ – 3’) | Product size (bp) |
| --- | --- | --- |
| Setd1b_ex5 (se) | CTGTTGGTGAGCTGGATGCTA | 172 bp |
| Setd1b_ex6 (as) | CTGGAGTAAGCTGTGTCTTGG |  |
| Rpl19 (se) | CTGATCAAGGATGGGCTGATC | 147 bp |
| Rpl19 (as) | CTTCTCAGGCATCCGAGCATT |  |
| Mpo (se) | CCTCGATGGAATGGGGAGAA | 165 bp |
| Mpo (as) | TCGAGGGTCTACTGAGTCGT |  |
| Klf-1 (se) | TACACCAAGAGCTCGCACCT | 145 bp |
| Klf-1 (as) | GACGATGTCCAGTGTGCTTC |  |
| Gata-1 (se) | AATGCCTGTGGCTTGTATCAC | 118 bp |
| Gata-1 (as) | TGGTCGTTTGACAGTTAGTGC |  |
| Il7r (se) | ACTCCAGAACCCAAGAATCAAG | 138 bp |
| Il7r (as) | AGGCCATACGACAGGTTTAATC |  |
| Ighm (se) | CGACTGGCTGAACCTGAATG | 189 bp |
| Ighm (as) | GCCAGGTTTGAGACCAGACA |  |
| Cebpa (se) | CAAAGCCAAGAAGTCGGTGGACAA | 150 bp |
| Cebpa (as) | TCATTGTCACTGGTCAACTCCAGC |  |
| PF-4 (se) | CTGGAGGTGATCAAGGCAGG | 102 bp |
| PF-4 (as) | ATATAGGGGTGCTTGCCGGT |  |
| c-Mpl (se) | GGAAGCTGTCTCGTCTCAGG | 215 bp |
| c-Mpl (as) | CTAGTGCGGTCTTGTTGCTG |  |
| Tek (se) | AAGGCACGCATCAAGAAGGA | 257 bp |
| Tek (as) | GCAGGGTCTGTCTCTAGCAC |  |
