# Supplementary material for: The H3K4 methyltransferase Setd1b is essential for hematopoietic stem and progenitor cell homeostasis in mice
Source: eLife. 2018 Jun 19;7:e27157. doi: 10.7554/eLife.27157 (PMC6025962; doi:10.7554/eLife.27157)
Supplement: Supplementary file 2 — (A) All antibodies and staining panels used for flow cytometry are depicted. (B) All antibodies and staining panels used for flow cytometry in transplanted mice are depicted. [file elife-27157-supp2.docx]

**Supplementary file 2A:** **FACS antibodies and staining panels**

| Surface  marker | Coupled dye | Clone | Dilution | Provider  (cat. number) |
| --- | --- | --- | --- | --- |
| **Lineage cocktail** | | | | |
| CD3ε | Biotin | 145-2C11 | 1:100 | eBioscience (13-0031) |
| CD4 | Biotin | GK1.5 | 1:800 | eBioscience (13-0041) |
| CD8α | Biotin | 53-6.7 | 1:200 | eBioscience (13-0081) |
| CD11b | Biotin | M1/70 | 1:400 | eBioscience (13-0112) |
| CD19 | Biotin | 1D3 | 1:400 | eBioscience (13-0193) |
| Gr-1 | Biotin | RB6-8C5 | 1:500 | eBioscience (13-5931) |
| NK1-1 | Biotin | PK136 | 1:800 | eBioscience (13-5941) |
| TCRβ | Biotin | H57-597 | 1:200 | eBioscience (13-5961) |
| TCRγδ | Biotin | GL-3 | 1:1600 | eBioscience (13-5711) |
| TER-119 | Biotin | TER-119 | 1:400 | eBioscience (13-5921) |
| **HSPC staining** | | | | |
| Lineage cocktail | as outlined above | | | |
| c-Kit | APC | 2B8 | 1:400 | eBioscience (17-1171) |
| Sca-1 | PE | D7 | 1:800 | eBioscience (12-5981) |
| CD34 | FITC | RAM34 | 1:25 | eBioscience (11-0341) |
| CD16/32 | PE-Cy7 | 93 | 1:800 | eBioscience (25-0161) |
| Flt3 | PerCP-eFluor710 | A2F10 | 1:100 | eBioscience (46-1351) |
| **Granulocyte and lymphocyte staining** | | | | |
| CD3ε | FITC | 145-2C11 | 1:100 | eBioscience (11-0031) |
| CD19 | PE-Cy7 | 1D3 | 1:800 | BD (552854) |
| Gr-1 | PE | RB6-8C5 | 1:400 | BD (553128) |
| CD11b | APC | M1/70 | 1:800 | eBioscience (17-0112) |

**Supplementary file 2B:** **FACS antibodies and staining panels (transplantation)**

| Surface  marker | Coupled dye | Clone | Dilution | Provider  (cat. number) |
| --- | --- | --- | --- | --- |
| **BM staining for transplantation (Figure 5)** | | | | |
| CD3ε | Biotin | 500A2 | 1:100 | BD (553239) |
| CD11b | Biotin | M1/70 | 1:500 | BD (553309) |
| CD11c | Biotin | HL3 | 1:400 | BD (553800) |
| CD19 | Biotin | 1D3 | 1:400 | BD (553784) |
| NK1-1 | Biotin | PK136 | 1:800 | eBioscience (13-5941) |
| Gr-1 | Biotin | RB6-8C5 | 1:1000 | eBioscience (13-5931) |
| TCRγδ | Biotin | GL-3 | 1:1600 | BD (553176) |
| TER-119 | Biotin | TER-119 | 1:400 | eBioscience (13-5921) |
| CD3ε | APC | 145-2C11 | 1:40 | BD (553066) |
| **CD45.1/CD45.2** **staining (granulocytes)** | | | | |
| CD45.1 | PE-Cy7 | A20 | 1:200 | eBioscience (25-0453) |
| CD45.2 | FITC | 104 | 1:400 | eBioscience (11-0454) |
| Gr-1 | PE | RB6-8C5 | 1:400 | BD (553128) |
| CD11b | APC | M1/70 | 1:800 | eBioscience (17-0112) |
| **CD45.1/CD45.2** **staining (lymphocytes)** | | | | |
| CD45.1 | PE | A20 | 1:200 | BD (561872) |
| CD45.2 | Alexa Fluor700 | 104 | 1:200 | BD (560693) |
| CD3ε | FITC | 145-2C11 | 1:100 | eBioscience (11-0031) |
| CD19 | PE-Cy7 | 1D3 | 1:800 | BD (552854) |
| **CD45.1/CD45.2** **staining (LSK)** | | | | |
| Lineage cocktail | as outlined in supplementary file 2A | | | |
| c-Kit | APC | 2B8 | 1:400 | eBioscience (17-1171) |
| Sca-1 | PE | D7 | 1:800 | eBioscience (12-5981) |
| CD34 | FITC | RAM34 | 1:25 | eBioscience (11-0341) |
| Flt3 | PerCP-eFluor710 | A2F10 | 1:100 | eBioscience (25-0161) |
| CD45.1 | PE-Cy7 | A20 | 1:200 | eBioscience (25-0453) |
| CD45.2 | Alexa-Fluor700 | 104 | 1:50 | BD (560693) |
| **CD45.1/CD45.2** **staining (LK)** | | | | |
| Lineage cocktail | as outlined in supplementary file 2A | | | |
| c-Kit | APC | 2B8 | 1:400 | eBioscience (17-1171) |
| Sca-1 | PE | D7 | 1:800 | eBioscience (12-5981) |
| CD34 | FITC | RAM34 | 1:25 | eBioscience (11-0341) |
| CD16/32 | PerCP-Cy5.5 | 93 | 1:200 | eBioscience (45-0161) |
| CD45.1 | PE-Cy7 | A20 | 1:200 | eBioscience (25-0453) |
| CD45.2 | Alexa-Fluor700 | 104 | 1:50 | BD (560693) |
